# A Developmental Timing Switch Promotes Axon Outgrowth Independent of Known Guidance Receptors

**DOI:** 10.1371/journal.pgen.1001054

**Published:** 2010-08-05

**Authors:** Katherine Olsson-Carter, Frank J. Slack

**Affiliations:** Department of Molecular, Cellular, and Developmental Biology, Yale University, New Haven, Connecticut, United States of America; Stanford University Medical Center, United States of America

## Abstract

To form functional neuronal connections, axon outgrowth and guidance must be tightly regulated across space as well as time. While a number of genes and pathways have been shown to control spatial features of axon development, very little is known about the *in vivo* mechanisms that direct the timing of axon initiation and elongation. The *Caenorhabditis elegans* hermaphrodite specific motor neurons (HSNs) extend a single axon ventrally and then anteriorly during the L4 larval stage. Here we show the *lin-4* microRNA promotes HSN axon initiation after cell cycle withdrawal. Axons fail to form in *lin-4* mutants, while they grow prematurely in *lin-4*–overexpressing animals. *lin-4* is required to down-regulate two inhibitors of HSN differentiation—the transcriptional regulator LIN-14 and the “stemness” factor LIN-28—and it likely does so through a cell-autonomous mechanism. This developmental switch depends neither on the UNC-40/DCC and SAX-3/Robo receptors nor on the direction of axon growth, demonstrating that it acts independently of ventral guidance signals to control the timing of HSN axon elongation.

## Author Summary

During development, nerve cells extend long structures called axons which are required for communication across distant brain regions and/or with other tissues. Many of the signals controlling the direction of axon extension are well understood, but much less is known about the factors that control when this growth occurs. Ultimately, failure to coordinate the direction and timing of axon elongation could lead to dramatic defects in the way our brains function. By studying the simple soil nematode *C. elegans*, we have identified mutations in three timing genes that result in either premature or delayed axon growth. These genes function independently from known directional signals as well as axon position. Since two of the genes are shared with mammals, findings presented here will help expand our understanding of human axon development, and could potentially lead to novel strategies for promoting growth in injured adult nerve cells.

## Introduction

During development, neurons must extend axons in the correct direction and at the proper time. Yet while several conserved families of ligands and receptors have been identified that control axon guidance [1], [2], little is known about the temporal regulation of axon growth. The observation that guidance cues can promote axon elongation as well as turning in *in vitro* experiments has led to models in which the timing of receptor gene expression and/or function specifies when axons extend [1]–[4]. However, it is clear that additional mechanisms must ensure that axon growth and guidance are appropriately coupled. For instance, in the *C. elegans* hermaphrodite specific neurons (HSNs), the UNC-40/DCC Netrin receptor is up-regulated at the L1 larval stage and ventrally localized by the L2, yet the HSNs do not extend single axons toward the ventral UNC-6/Netrin source until early L4, nearly two stages later [5]. In addition, HSN axons grow at the correct stage in animals lacking UNC-6/Netrin or UNC-40/DCC, suggesting that the timing of axon elongation is regulated independently of Netrin signaling [5].

The *C. elegans* HSNs provide a convenient system for further investigating the temporal control of axon elongation. Many of the principle molecules required for *C. elegans* axon growth and guidance are conserved in higher animals [5]–[10]. In addition, the morphological features of HSN development have been very well characterized [5]. At the mid-to-late L3 larval stage, the neurons extend several short neurites ventrally, and at the L3/L4 transition, they retract all but one of these processes, the neurite selected to become the axon. In the L4, the axon completes its extension toward the ventral nerve cord (VNC) and turns anteriorly, where it forms synapses with vulval muscles and the VC4 and VC5 motor neurons before growing to the nerve ring [5], [11].

We predicted that genes which regulate the timing of cell divisions might also control HSN postmitotic differentiation. The *C. elegans* transcription factor *lin-14* has previously been shown to play a similar role during synaptic development of the DD motor neurons [12]–[14]. *lin-14* is a member of the heterochronic pathway, a set of temporal patterning genes that was first characterized in mitotic hypodermal cells [15]–[18]. Several other heterochronic genes are also expressed in the *C. elegans* nervous system, including those encoding the *lin-4* microRNA, the cytoplasmic RNA-binding protein LIN-28, nuclear hormone receptor DAF-12 and the HBL-1 transcription factor [19]–[25]. However, the neuronal functions and interactions of these genes with *lin-14*, if any, have not yet been determined.

MicroRNAs (miRNAs) represent a set of non-coding RNAs that inhibit genes post-transcriptionally by binding to partially complementary mRNA sequences [19], [20], [26], [27]. While they are dynamically expressed during the development of invertebrate and vertebrate nervous systems, only a small number have been characterized *in vivo* [28]–[31]. Here, we show that the *lin-4* miRNA together with *lin-14* and *lin-28* comprise a cell-autonomous switch that promotes axon growth. These genes act independently of ventral guidance receptors to ensure that axon development is properly completed before the HSNs are required in the adult egg-laying system [32].

## Results

### The *lin-4* miRNA promotes HSN axon elongation

**Figure 1:**
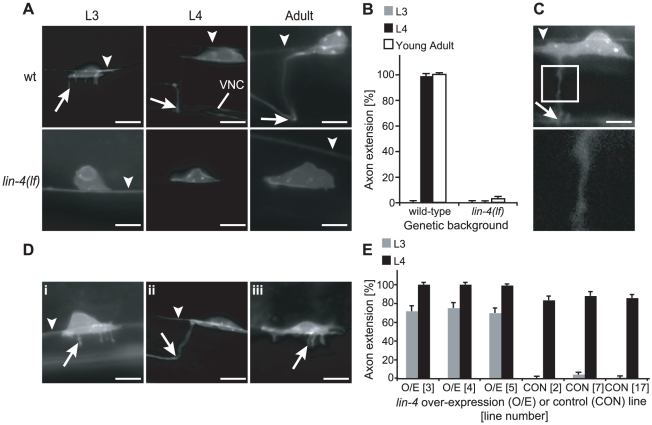
*lin-4(lf)* displays delayed HSN axon extension. (A) In late L3 wild-type (wt) animals (top), HSNs extended multiple neurites (arrow) in the ventral direction (left). By early L4 (middle) and in the adult (right), wild-type animals extended a single HSN axon ventrally and then anteriorly. VNC: ventral nerve cord. In L3, L4, and adult *lin-4(lf)* animals (bottom), no neurites or axons were typically seen. (B) Percentage of L3, L4, or adult wild-type or *lin-4(lf)* animals in which HSN axons had completed their anterior turn. n≥100 for each time point. (C) Image of one of the few *lin-4(lf)* adult animals with axon outgrowth (top). When the region contained by the box is magnified (bottom), variation in axon diameter is clearly visible. (D) Cell-autonomous over-expression of the *lin-4* O/E construct led to precocious neurite outgrowth in early L3 (i) and axon elongation by late L3 (ii), while over-expression of the control construct resulted in neurite outgrowth, but not axon elongation, by late L3 (iii). (E) Percentage of L3- or L4-stage animals with axon extension in *lin-4* over-expression (O/E) or control (CON) lines. All *lin-4* O/E (Lines 3, 4, and 5) or control (Lines 2, 7, and 17) strains contained the integrated *unc-86::myr-GFP* reporter to visualize axon outgrowth. n≥50 for each time point. In A, C and D, the arrowheads point to the PLM axon, scale bars represent 5 µm, and anterior is depicted to the left and ventral is down. In A (wt L3), Di and Diii, the arrow points to one of several neurites, and in A (wt L4 and Adult), C, and Dii, it refers to the HSN axon's anterior turn. For B and E, error bars represent standard error of proportion.

Candidate heterochronic mutants were crossed into a transcriptional reporter strain expressing myristoylated green fluorescent protein (GFP) under the control of the *uncoordinated-86 (unc-86)* promoter [5]. *unc-86* encodes a POU homeodomain transcription factor that is expressed constitutively after HSNs exit the cell cycle and controls execution of late maturation events [33], [34]. In this work, the phenotypic criterion for distinguishing HSN axons from less mature neurites was initiation of the anterior turn (Figure 1A).

Loss-of-function (lf) mutants for the *lin-4* miRNA displayed a penetrant retarded phenotype, in which HSN axons were not detected in L3 or L4 animals or the majority of young adults scored (Figure 1A and 1B). Sporadic neurite outgrowth was observed in *lin-4(lf)* HSNs after mid-L4, but was distinct from the extensive neurite outgrowth patterns that precede axon extension in wild-type animals (Figure 1A). In the few young adults with detectable axon outgrowth, processes were often morphologically abnormal (Figure 1C). Since a percentage of older adults also extended axons (data not shown), it is likely that this *lin-4(lf)* phenotype represents a developmental timing defect and not simply a uniform inability to initiate growth.

We predicted that if *lin-4* acts as a temporal regulator of axon development, then *lin-4* over-expression would lead to precocious axon elongation, opposite to the phenotype observed in *lin-4(lf)* mutant animals. MiRNAs can be ectopically expressed *in vivo* using cell- or tissue-specific enhancers or promoters [35], and ∼80 nucleotides (nt) of both upstream and downstream sequences have been shown to be sufficient for processing *in vitro* [36]. To over-express *lin-4* in the HSNs, the *lin-4* hairpin and ∼160 nt of total flanking sequence were sub-cloned behind the *unc-86* promoter, and the resulting construct was injected into wild-type N2 animals. Control lines were generated using the same construct as described above, but with the 21 nt mature *lin-4* sequence deleted.

Three lines that express these *lin-4* or control constructs were scored for timing of HSN axon extension. As would be expected if *lin-4* specifically regulates HSN temporal patterning, animals that over-expressed *lin-4* exhibited precocious neurite and axon outgrowth during L3 (Figure 1Di, 1Dii and 1E), while animals expressing the control construct selected axons at the same time as observed in wild-type (Figure 1Diii and 1E).

### The heterochronic genes *lin-14* and *lin-28*, but not *daf-12*, inhibit axon extension in the HSNs

In the *C. elegans* hypodermis, *lin-4* controls the execution of L1- and L2-stage cell division patterns by inhibiting *lin-14* and *lin-28*, respectively [19], [20], [24]. Seven *lin-4* complementary elements (LCEs) have been identified in the *lin-14* 3′UTR and one has been found in *lin-28*, consistent with both genes being direct targets of *lin-4* [37]. Unlike *lin-14*, *lin-28* is conserved in *Drosophila* and vertebrates, and along with the *lin-4* homolog miR-125, is widely expressed in the vertebrate nervous system [30], [31], [38]–[40]. LIN-28 has also been shown to be one of four ‘stemness’ factors that are capable of reprogramming differentiated mouse fibroblasts to a pluripotent stem cell state, in part by inhibiting processing of the *let-7* pri-miRNA [38], [41], [42].

**Figure 2:**
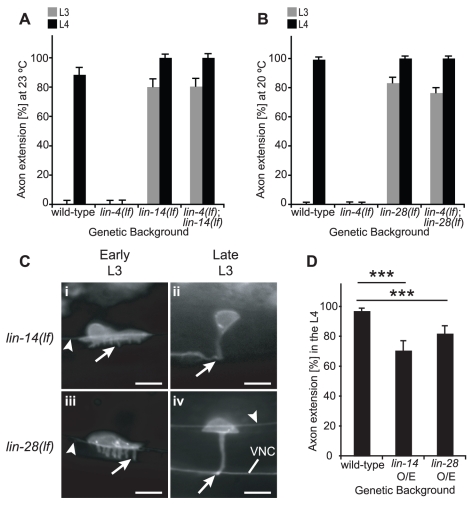
*lin-14* and *lin-28* control the timing of HSN axon extension. (A) Single mutant *lin-14(lf)* animals extended axons precociously, and in double *lin-4(lf)*; *lin-14(lf)* mutants, *lin-14(lf)* was sufficient to suppress the *lin-4(lf)* retarded phenotype at the restrictive temperature (23°C). n≥50 for all time points. (B) *lin-28(lf)* animals displayed precocious axon outgrowth, and *lin-28(lf)* completely suppressed the *lin-4(lf)* retarded phenotype. n≥100 for all conditions. (C) Representative HSNs from *lin-14(lf)* (i–ii) or *lin-28(lf)* (iii–iv) early L3 animals with precocious outgrowth of multiple ventral neurites (arrow, i,iii) or late L3-stage animals with precocious axon outgrowth (arrow, ii,iv). Arrowhead: the PLM axon. VNC: ventral nerve cord. Animals are depicted with anterior to the left and ventral down, and scale bars represent 5 µm. (D) Over-expression of *lin-14* or *lin-28* led to a significant delay in axon extension during the L4 stage. ***: p<0.001 for the difference between wild-type and either *lin-14* or *lin-28* O/E using the two-sample z-test. n≥50 for each strain. For (A, B, D), error bars represent standard error of proportion.

**Figure 3:**
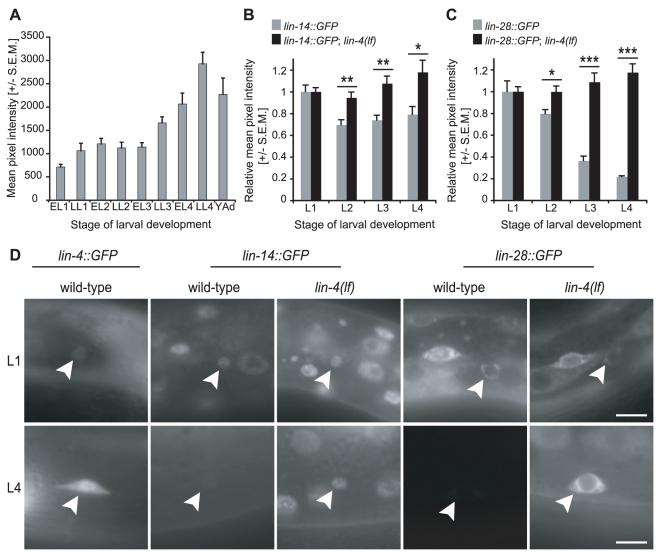
*lin-4* and its targets *lin-14* and *lin-28* are expressed reciprocally during larval development. (A) In an integrated *lin-4::GFP* reporter strain, GFP is up-regulated in the HSNs during larval development. The y-axis depicts raw average pixel intensity values, and n = 10 for each time point. Note that the change in GFP expression levels between L1 and Adult stages exceeded the dynamic range of the camera, and at the set exposure time, only pixel intensity values for L1, L2, and L3 were always within the linear range. Some images acquired for late L4 and young adult stages were oversaturated, and thus the changes in pixel intensity after L3 are likely to be under-representations of the true fold increases in GFP expression. EL1–4: Early Larval Stage 1–4. LL1–4: Late Larval Stage 1–4. YAd: Young Adult. (B) GFP expression from an integrated *lin-14::GFP* reporter was down-regulated in the HSNs by the L2 stage, while in *lin-4(lf)* mutants, GFP expression was maintained. **p-values≤0.01 (0.005 for L2 and 0.003 for L3). *p-value = 0.015. (C) A *lin-28::GFP* reporter strain (Line 10-2) was down-regulated in the HSNs during L2 and L3 stages. When it was crossed into *lin-4(lf)*, GFP expression persisted throughout larval development. *p-value = 0.012. ***p-values<0.001 (2.5×10^−5^ for L3 and 6.3×10^−7^ for L4). For B and C, n≥10 for each time point. Note that for each strain, pixel intensity values for L2, L3, and L4 were normalized to average L1 values and do not represent absolute fluorescence intensities. In addition, the *lin-14::GFP* strain expressed GFP more weakly than the *lin-28* reporter during the L1, and thus a much smaller decrease in relative pixel intensity was required for complete down-regulation of the *lin-14::GFP* transgene. p-values for differences in relative pixel intensity were obtained using the two-sample t-test. In (A–C), all error bars represent standard error of the mean (S.E.M.). (D) Representative images of HSNs from L1 (top) and L4 (bottom) animals bearing *lin-4*, *lin-14*, or *lin-28* GFP reporters in wild-type or *lin-4(lf)* backgrounds as noted. Arrowhead: HSN. Scale bars represent 5 µm, and anterior is to the left and ventral is down.

In animals with loss-of-function mutations in *lin-14* or *lin-28*, axons were detected prematurely during the L3 stage (Figure 2A, 2B, 2Cii, and 2Civ). Neurite outgrowth was also precocious (Figure 2Ci and 2Ciii), indicating that these phenotypes are unlikely to be due to dramatic changes in rate of elongation or axon selection. By contrast, HSN axon guidance was generally unaffected, although *lin-14(lf)* HSNs exhibited failed ventral and/or anterior growth on occasion (data not shown). To test whether *lin-14* and *lin-28* act to inhibit axon extension, each gene was over-expressed using translational fusion constructs known to be down-regulated during larval development ([24], [43]; Figure 3B and 3C). Retarded axon elongation was indeed observed (Figure 2D), yet phenotypes may have been more pronounced in transgenic animals constitutively expressing *lin-14* or *lin-28*. In support of this prediction, loss of the *lin-4* target sites in a *lin-14* gain-of-function (gf) mutant resulted in severe axon growth delays [15.9% L4 animals with axons (n = 82)], with significant differences in percentage outgrowth between *lin-14(gf)* and either wild-type or *lin-14::GFP*-expressing animals (p<0.0001 for each comparison using the two-sample z-test) [44], [45].

Previously, *lin-14* loss-of-function mutations were found to be more robust in suppressing *lin-4(lf)* hypodermal phenotypes than those for *lin-28* [15], [46], suggesting that in the hypodermis, *lin-4* interacts more strongly with *lin-14*. In addition, loss-of-function alleles for *lin-14*, but not *lin-28*, were sufficient to restore vulval development and dauer formation in *lin-4(lf)* mutants [46], making it likely that *lin-14* genetically interacts with *lin-4* in greater subset of cells. To test whether *lin-14* and *lin-28* function with *lin-4* to control HSN axon outgrowth, *lin-4(lf)*;*lin-14(lf)* and *lin-4(lf)*;*lin-28(lf)* double mutants expressing the *unc-86::myr-GFP* reporter were scored for axon extension. Strikingly, the presence of either *lin-14(lf)* or *lin-28(lf)* almost completely suppressed *lin-4(lf)*, resembling the observations for *lin-14(lf)* and *lin-28(lf)* single mutants (Figure 2A and 2B).

The *daf-12* nuclear hormone receptor has been shown to interact with *lin-14* and *lin-28* in the hypodermis, and a gain-of-function allele of the gene leads to retarded seam cell development [25], [47], [48]. Recently, *daf-12* was also found to regulate the extension of *C. elegans* muscle arms, membrane protrusions that are required for forming productive contacts between muscles and motor neurons [49]. We tested whether the DAF-12 receptor could also play a role in the HSN axon growth response, but failed to detect changes in the timing of elongation in gain-of-function (*rh61*) or null (*rh61 rh411*) alleles. As seen in wild-type, no axons extended during the L3 in either mutant (n = 51 for *rh61* and n = 60 for *rh61rh411*) while nearly all displayed outgrowth in the L4 [98.1% (n = 52) for *rh61* and 94.3% (n = 53) for *rh61rh411*]. In addition, when we crossed the *daf-12* null mutant into *lin-4(lf)*, we did not observe axon extension at the L4 (n = 54) or adult (n = 25) stages, indicating that in contrast to *lin-14* and *lin-28*, *daf-12* does not act downstream of *lin-4* to control HSN axon growth.

### The *lin-4* miRNA and both *lin-14* and *lin-28* are reciprocally expressed in the HSNs

The expression pattern of *lin-4* in the HSNs was determined using an integrated *lin-4::GFP* reporter strain [50]. GFP was first detected in the late L1 and values progressively increased in later stages until they spiked in the L4 (Figure 3A and 3D). These data highlight the dynamic tissue-specific changes in *lin-4* expression and contrast with the pattern determined by northern analysis using RNA from whole animals, in which *lin-4* was up-regulated in the late L1 and then remained at relatively constant levels through the remainder of larval development [51].

To characterize *lin-14* and *lin-28* expression, GFP was quantified in the HSNs in strains containing integrated *lin-14::GFP* or an extrachromosomal array of *lin-28::GFP*. Both reporter constructs were previously shown to rescue *lin-14* and *lin-28* mutant phenotypes, respectively, and the *lin-14::GFP* transgene was found to display expression patterns that were consistent with results from anti-LIN-14 antibody staining [24], [43]. In the HSNs, *lin-14* and *lin-28* displayed reciprocal expression patterns to *lin-4*, in which they were expressed at their highest levels in the L1, and became undetectable in later larval stages (Figure 3B–3D). To test the possibility that *lin-4* was required for their down-regulation, the *lin-4(lf)* mutant allele was crossed into the *lin-14* and *lin-28* reporter lines, and GFP levels were quantified over time. When *lin-4* was absent, expression of *lin-14* and *lin-28* was maintained during larval development, confirming that *lin-4* is indeed necessary for their down-regulation in the HSNs (Figure 3B–3D).

### The regulation of *lin-28* by *lin-4* and *lin-14* changes over developmental time

In the *C. elegans* hypodermis, *lin-14* is confirmed to be a direct *lin-4* target, while the extent to which *lin-28* is regulated by *lin-4* through its single LCE is still unclear [19], [20], [24]. In one study, deletion of the LCE in a *lin-28* translational reporter resulted in the maintenance of hypodermal gene expression during late larval stages, demonstrating that this site is important for *lin-28* down-regulation [24]. However, removal of three nucleotides from the LCE resulted in only a modest increase in expression of a transgene containing a hypodermal promoter fused to lacZ and the *lin-28* 3′UTR [47]. Several additional cis regulatory elements as well as trans-acting factors, including the protein LIN-66, have now been shown to be important for regulation of *lin-28* expression in the hypodermis [47], [48].

**Figure 4:**
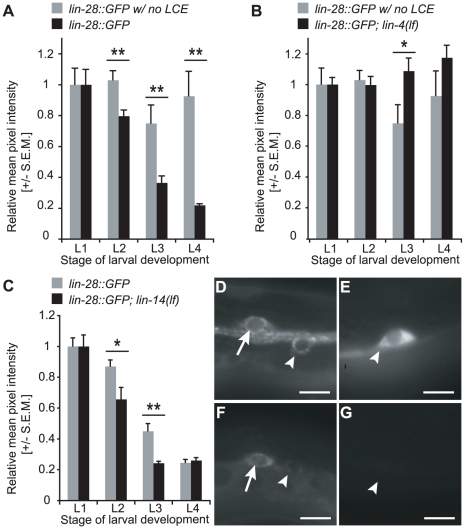
Regulation of *lin-28* by *lin-4* and *lin-14* changes over developmental time. (A) Relative GFP levels were higher for *lin-28::GFPΔLCE* (Line 5-1) lacking the LCE than for *lin-28::GFP* (Line 10-2) with an intact 3′UTR at the L2, L3, and L4 stages. **p-values≤0.01 (0.005 for L2, 0.01 for L3, and 0.002 for L4). (B) Relative GFP intensity was lower at the L3 stage in *lin-28::GFPΔLCE* (Line 5-1) lacking the LCE than in *lin-28::GFP* (Line 10-2) crossed into *lin-4(lf)*. *: p-value = 0.030. For A–B, changes in LIN-28::GFP levels across larval development in animals lacking the LCE or *lin-4* exceeded the dynamic range of the camera. At the set exposure time, some images acquired for late L4 were oversaturated. n≥10 for each strain and time point. (C) Relative *lin-28::GFP* (Line 10-2) expression was lower in L2- and L3-stage HSNs in *lin-14(lf)* compared to wild-type at 23°C. n≥9 for each *lin-28::GFP* condition and n≥18 for each *lin-14(lf)*; *lin-28::GFP* condition. *: p-value = 0.019. **: p-value = 0.001. For A–C, data were normalized to L1 values for each strain, p-values for differences in relative pixel intensity were obtained using the two-sample t-test, and error bars represent S.E.M. (D,E) Representative L1- (D) and L4-stage (E) HSNs from *lin-28::GFPΔLCE* (Line 5-1). (F,G) Representative L1- (F) and L4-stage (G) HSNs from *lin-28::GFP* (Line 10-2); *lin-14(lf)*. For (D–G), scale bars represent 5 µm, and anterior is left and ventral is down. Arrowhead: HSN. Arrow: CAN neuron.

To investigate whether *lin-4* targets *lin-28* directly in the HSNs, the changes in GFP expression relative to L1 values were determined for a *lin-28::GFP* transgenic line in which the LCE was deleted (Figure 4A, 4B, 4D, and 4E). Consistent with a direct regulatory role for *lin-4*, relative GFP levels were elevated in the absence of the LCE compared to the control (Figure 4A, 4D, and 4E). However, in contrast to the pattern observed in *lin-4(lf)* mutants, LIN-28::GFP expression partially declined in the L3 stage (Figure 4B). This trend was also observed in a second *lin-28::GFPΔLCE* line (data not shown), suggesting that *lin-4* also regulates *lin-28* indirectly through an LCE-independent mechanism.

In the hypodermis, *lin-14* is required for maintenance of *lin-28* expression in a *lin-4(lf)* mutant background [24]. To test whether it is also a positive regulator of *lin-28* in the HSNs, GFP expression was quantified in *lin-14(lf)*; *lin-28::GFP* animals that were maintained at the restrictive 23°C temperature (Figure 4C, 4F, and 4G). Introduction of the *lin-14* mutant allele resulted in a significant decrease in GFP expression in the HSNs at the L2 and L3 stages, leading to complete down-regulation of the reporter one stage earlier than in wild-type animals grown under the same conditions (Figure 4C, 4F, and 4G). This result indicates that one mechanism by which *lin-4* indirectly controls *lin-28* expression in the HSNs is through the down-regulation of *lin-14*.

### *lin-4* functions cell-autonomously to control HSN axon elongation

Since *lin-4* was found to be expressed and to genetically interact with *lin-14* and *lin-28* in the HSNs, it likely acts cell autonomously to regulate the timing of axon development. To test this possibility directly, transgenic lines over-expressing *lin-4* under the control of the *unc-86* promoter (Figure 1D and 1E) were crossed into *lin-4(lf)* and scored for rescue of the *lin-4* retarded phenotype. The *unc-86* promoter has previously been used to test autonomous function or drive transgene expression in the HSNs [5], [52], [53], in large part because it is not expressed in neighboring tissues, including the ventral nerve cord or cells of the egg-laying system.

**Figure 5:**
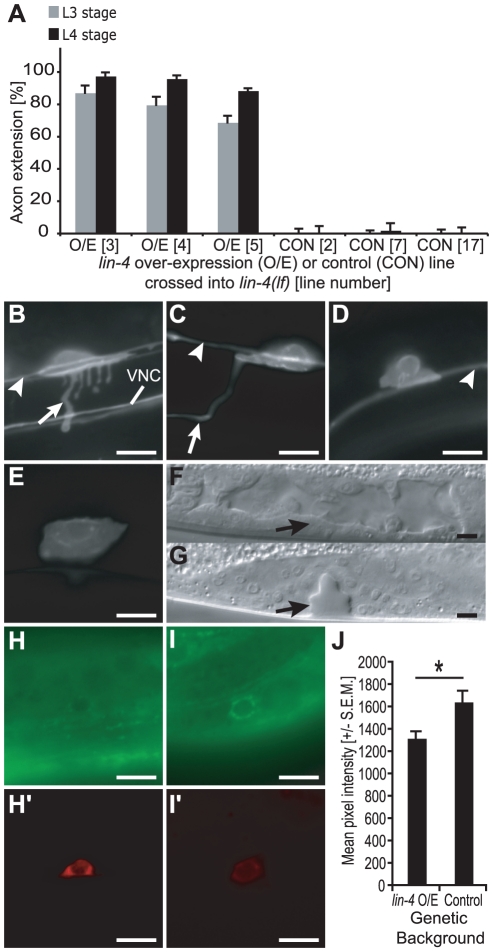
*lin-4* regulates HSN axon extension cell autonomously. (A) Strains containing the *lin-4(lf)* mutation and the integrated *unc-86::myr-GFP* reporter were crossed into the *lin-4* over-expression (Lines 3, 4, and 5) or control (Lines 2, 7, and 17) strains, and L3 and L4 animals were scored for axon outgrowth. The percentage of animals with axon extension in at least one HSN is shown, with n≥50 for each stage. Error bars represent standard error of proportion. (B,C) In animals with the *lin-4* over-expression construct, multiple ventral neurites were detected in HSNs in early L3 (B, arrow), and axon outgrowth was visible in the mid-to-late L4 (C, arrow). VNC: ventral nerve cord. (D,E) In animals expressing the control construct, no neurites or axons were detectable at the L3 (D) or L4 (E) stages. In (B–D), arrowhead: PLM axon. (F) In the animal depicted in C, the *lin-4(lf)* vulvaless phenotype was not rescued and vulva development was not observed (arrow). (G) Developing vulva (arrow) from wild-type mid-to-late L4 animal. (H–J) The *unc-86* promoter was used to drive over-expression of the *lin-4* hairpin or a control lacking the mature *lin-4* sequence in the HSNs. Representative *lin-4* over-expression (O/E) (Line 4) and control (Line 7) strains were crossed into a *lin-28::GFP* sensor carrying its native 3'UTR (Line 10-2). In both the *lin-4* O/E and control lines, HSNs inheriting the transgenes expressed the dsRED2 marker. (H–I′) Representative images of HSNs expressing either the *lin-4* O/E (H and H′) or control (I and I′) construct, acquired with GFP(BP) (H and I) or TRITC (H′ and I′) filters. The scale bars in B–I′ represent 5 µm, and anterior is to the left and ventral is down. (J) The mean pixel intensity in 10 L2-stage HSNs from the *lin-4* O/E and control strains. *: p-value = 0.021 using a two-sample *t*-test for the difference between two means. Error bars represent S.E.M.

Precocious HSN axon extension was observed for all three lines, likely due to the predicted over-expression of *lin-4* from the multi-copy transgene (Figure 5A–5C). In addition, removal of the mature *lin-4* sequence from the rescuing construct resulted in a failure to restore axon extension in *lin-4(lf)* mutants (Figure 5A, 5D, and 5E), demonstrating that the effect was specific. This rescue of retarded axon growth was unlikely to be due to extrinsic signaling from other *unc-86*-expressing cells like the PLMs (depicted in Figure 1, Figure 2, and Figure 5; [11], [34], [54]). Precocious HSN axon outgrowth was still observed upon *lin-4* over-expression when HSNs were dorsally displaced relative to the PLMs, such as in *unc-40(e721)* mutants (data not shown; [5]). Rescue was also likely not due to the export of *lin-4* to adjacent cells, since the *lin-4(lf)* vulvaless defect persisted in animals expressing *unc-86::lin-4* (Figure 5F and 5G; [26]).

To confirm independently that functional *lin-4* is generated in the *lin-4* over-expression (O/E) lines, representative O/E (Line 4) and control (Line 7) lines were crossed into a *lin-28::GFP* sensor (Line 10-2) whose down-regulation is known to be *lin-4*-dependent (Figure 3C and 3D). A comparison of average pixel intensity values in L2-stage HSNs showed that GFP levels were lower in the *lin-4* O/E line compared to the control, as would be expected if functional *lin-4* were expressed in the HSNs (Figure 5H–5J).

### Precocious axon outgrowth is not dependent on *unc-40/DCC* or *sax-3/Robo*

Axon guidance factors not only control the direction of growth cone migrations, but they also promote axon outgrowth [3]. In the HSNs, the Netrin receptor UNC-40/DCC is known to be required cell-autonomously for cellular polarization as well as for directing ventral axon extension [5]. UNC-40 is up-regulated during the late L1 stage and by L2 is localized primarily to the ventral surface in response to the release of UNC-6/Netrin from the VNC [5], [55]. Interestingly, HSN axons extend two larval stages after this Netrin response [5], suggesting that Netrin signaling alone is not sufficient to induce growth. This conclusion is further corroborated by the finding that HSN axons are misguided but elongate at the appropriate time in mutants for *unc-6*/netrin or *unc-40*/DCC [5].

**Figure 6:**
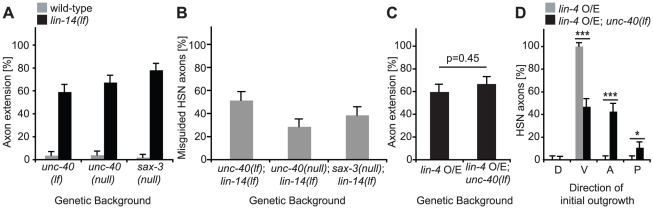
Precocious axon outgrowth is not dependent on proper guidance. (A) Strains containing strong loss-of-function (lf) or null alleles of *unc-40* and *sax-3* were scored for timing of axon outgrowth at the L3 larval stage in the presence or absence of *lin-14(lf)*. At the restrictive temperature (23°C), *lin-14(lf)* animals continued to extend axons precociously in *unc-40(lf)*, *unc-40(null)*, or *sax-3(null)* mutant backgrounds. By contrast, single *unc-40* or *sax-3* mutants did not display marked outgrowth at the L3 stage. (B) HSN axons scored in A were categorized as misguided if they failed to extend initially in the ventral direction. (C) L3-stage animals expressing an integrated *lin-4* over-expression (O/E) construct displayed precocious axon extension. There was no statistically significant change in outgrowth (p = 0.45) in the presence of *unc-40(lf)*. (D) The predominant initial direction of growth for HSN axons scored in (C) were categorized as dorsal (D), ventral (V), anterior (A), or posterior (P). *p-value = 0.038. ***p-values are both <0.0001. For (A,C), the percentage of animals with axon extension in at least one HSN is shown, with n≥50 for each stage. For (C,D), p-values were determined using the two-sample z-test. For (A–D), error bars represent standard error of proportion.

To determine whether the *lin-4* switch functions as part of this Netrin-independent timing mechanism, *lin-14(lf)*; *unc-86::myr-GFP* animals were crossed with animals containing either a severe loss-of-function (*e271*) or null (*e1430*) allele of *unc-40* [6], [43], [56]. In both double mutants, precocious axon outgrowth was observed at the L3 stage (Figure 6A). Premature axon extension was also observed in *lin-14(lf)* animals lacking functional SAX-3/Robo, a receptor which acts in parallel to UNC-40 to promote ventral growth in the HSNs (Figure 6A) [57]. While guidance defects for *unc-40(e271)*, *unc-40(e1430)*, and *sax-3(ky123)* were incompletely penetrant as previously described [5], [57], the precocious phenotypes observed in all three double mutants were not confined to those animals with intact ventral outgrowth (Figure 6B).

Since our genetic and expression data demonstrated that *lin-4* acts upstream of *lin-14*, we predicted that *lin-4* – like *lin-14* – also controls the timing of axon growth independent of ventral guidance. To confirm this genetically, *unc-40(e271)* mutants were crossed into a strain expressing the integrated *unc-86::lin-4* over-expression vector and the *unc-86::myr-GFP* reporter. No statistical difference in the percentage of animals extending axons precociously was observed between wild-type and *unc-40(e271)* (Figure 6C), despite the fact that there was a dramatic reduction in the number of HSNs that extended axons ventrally in the *unc-40(e271)* background (Figure 6D). Precocious growth was also seen in HSNs that exhibited defects in ventral cell migration, another hallmark of *unc-40(lf)* mutants (data not shown; [5]). Based on these findings, we concluded that the *lin-4* developmental switch acts independently of ventral guidance signals to control the timing of HSN axon extension.

## Discussion

### The *lin-4* miRNA cell-autonomously controls the timing of HSN axon elongation after cell cycle exit

In the nervous system, the timing of the birth of neurons is critical for their specification [58]–[62]. In specific neuronal lineages, this is likely due to the coupling of terminal cell divisions with distinct differentiation programs [63], [64]. However, evidence is emerging that at least in the vertebrate neocortex, determination of laminar identity is not simply controlled by the cell cycle [65], [66].

In the HSNs, the temporal regulation of axon outgrowth by *lin-4* also appears to be regulated postmitotically. In wild-type animals, *lin-4* is initially detected in the HSNs during the first larval stage (Figure 3A), well after the neurons exit the cell cycle during embryogenesis [33]. Moreover, in *lin-4(lf)* mutants, HSNs are still born, express the neuronal determination gene *unc-86*, and complete their anterior migration prior to L1 [34], [54], [67], confirming that *lin-4* is only required for maturation events that occur after it is normally up-regulated. Finally, the postmitotic expression of *lin-4* under the *unc-86* promoter [34], [54] rescues the *lin-4(lf)* retarded axon growth phenotype, strongly suggesting that *lin-4* functions cell-autonomously after cell cycle withdrawal. While the *lin-4* site of action has previously been predicted for a number of cell types, it has previously not been verified *in vivo* [19], [20], [24], [47], [68].

### LIN-14 and LIN-28 inhibit HSN maturation

The *lin-4* miRNA is required for the down-regulation of *lin-14* and *lin-28* in the HSNs. Over-expression of each *lin-4* target leads to a delay in HSN axon outgrowth, and when LIN-14 and LIN-28 levels are maintained in *lin-4(lf)* mutants, HSN axon extension is not detected during larval development. A loss-of-function mutation in either gene suppresses the *lin-4(lf)* delay and results in axons extending ∼one stage too early. Strikingly, similar one-stage shifts in the timing of a later differentiation event, the expression of the serotonin-synthesis gene *tph-1*, are also observed in *lin-14(lf)* and *lin-28(lf)* mutants in the presence or absence of the *lin-4* loss-of-function allele (Figure S1). Since the relative timing of axon outgrowth and *tph-1* expression is unaltered in these animals, it is likely that after axon initiation the overall rate and sequence of differentiation remains unchanged.

It has been proposed that in hypodermal lineages, the transcription factor LIN-14 specifies L1-stage cell divisions, while LIN-28 acts post-transcriptionally to specify L2 and/or L3 developmental events [13], [37]. Limited examples from the *C. elegans* nervous system suggest that *lin-14* and possibly *lin-28* may function similarly in postmitotic cells. In the HSNs, LIN-14 and LIN-28 expression patterns are consistent with roles in preventing the progression to L2 or L3/L4 fates, respectively (Figure 3B–3D). *lin-14* is also required during the L1 stage to inhibit precocious synaptic remodeling in the DD-type motor neurons as well as for expression of the ventral cord maintenance factor *zig-4* in the PVT interneuron [14], [69]. Interestingly, *lin-28* is not necessary for *zig-4* expression [69], potentially because it is required to promote L2- and/or L3-specific temporal identities. Further studies will be required to confirm this possibility, as well as to determine whether *lin-14* and *lin-28* interact with *lin-4* or other known heterochronic genes—such as *hbl-1* or the *let-7* miRNA—in these or other neurons [22], [23], [70]. However, given that the heterochronic gene *daf-12* functions with *lin-14* and *lin-28* in the hypodermis [25], [48] but does not control axon growth in the HSNs, it is clear that there is no universal mechanism governing temporal patterning across tissues.

### The interactions between the *lin-4* microRNA and its targets depend on cellular and developmental context

Although *lin-4* functions together with two previously identified targets – *lin-14* and *lin-28* – in the HSNs, its interactions with these genes are distinct from patterns described for other cell types. In the *C. elegans* hypodermis, *lin-14(lf)* is a more efficient suppressor of *lin-4(lf)* than *lin-28(lf)*, and in the vulva, only *lin-14(lf)* is able to suppress the *lin-4(lf)* vulvaless defect [15], [46]. In the HSNs, by contrast, both *lin-14(lf)* and *lin-28(lf)* can strongly suppress the delays in axon outgrowth observed in *lin-4(lf)* mutants.

The interactions between *lin-4* and its targets are highly dynamic in the HSNs, and the importance of the LCE in *lin-28* down-regulation—which potentially reflects the role of direct *lin-4* binding—also varies over developmental time. After the L2 stage, for instance, relative levels of LIN-28 are lower when the LCE is removed than in *lin-4(lf)*, revealing that *lin-4* likely inhibits *lin-28* through both direct and indirect mechanisms. The latter is mediated at least in part by *lin-14*, since *lin-14* is targeted by *lin-4* and promotes *lin-28* expression in the HSNs. Ultimately, the differences in the interactions between *lin-4*, *lin-14*, and *lin-28* at distinct postembryonic stages of HSN development demonstrate the importance of tracking miRNA regulation in its *in vivo* context, and reveal the limitations of identifying and functionally characterizing miRNA targets in isolated cell culture systems.

### The *lin-4* switch controls timing of HSN axon outgrowth independent of known ventral guidance receptors

We have demonstrated that up-regulation of the *lin-4* miRNA and down-regulation of its targets *lin-14* and *lin-28* are required for axon initiation in the HSNs. Previous work has shown that axons grow in response to extracellular matrix proteins, guidance cues, and/or trophic factors [1]–[4], [9], [71], [72]. It is therefore likely that the *lin-4* switch functions by altering the expression, activity and/or localization of the receptors that detect these signals or by inhibiting downstream cytoskeletal remodeling events until the appropriate developmental time.

While the intracellular targets of the *lin-4* switch have not yet been identified, they appear to act independently from the UNC-6/Netrin and SLT-1/Slit ventral guidance pathways. We continued to observe temporal shifts in HSN axon elongation in mutants for the Netrin and Slit receptors, and these shifts did not require ventral growth, the initial direction of HSN axon elongation in wild-type animals [5]. Conversely, precocious axon initiation in *lin-14(lf)*, *lin-28(lf)*, and *lin-4* O/E animals had little effect on pathfinding, likely because outgrowth still occurs well after *unc-6/*netrin and *slt-1/*slit are normally up-regulated and have been detected by the HSNs [5], [55], [57]. In light of these observations, it will be interesting to determine whether at least a subset of uncharacterized axonal wiring genes act by ensuring the proper timing of growth relative to specific guidance responses during development.

### Conclusions

In this work, we have described a cell-autonomous timer that is activated by the *lin-4* miRNA after embryonic development. In *lin-4(lf)* mutant animals, neurons fail to extend axons prior to the larval-to-adult transition, and axons in the adult are morphologically abnormal. Thus, *lin-4* is necessary to ensure that HSN axon development is properly completed before the neurons are required in the adult egg-laying circuit [33].

In the future, it will be worthwhile to apply these findings to mature cells, and test whether misexpression of *lin-4* or its targets is sufficient to reset intrinsic developmental clocks and/or reinitiate postmitotic maturation events, including axon elongation. Since *lin-4* and *lin-28* are conserved in higher animals and are also expressed in *in vitro* models of neuronal differentiation [73], [74], studies such as these could potentially lead to new regenerative strategies for treating diseases or injuries within the vertebrate nervous system.

## Materials and Methods

### Nematode strains

All animals were maintained at 20°C using standard protocols as previously described [75], unless indicated otherwise. For experiments which included temperature-sensitive *lin-14(lf)* animals, all strains were transferred to 23°C as gravid adults and phenotypes were scored in progeny.

The wild-type strain was N2 Bristol, and genetic mutant alleles included *lin-4(e912)*, *lin-14(n179ts)*, *lin-14(n355gf)*, *lin-28(n719)*, *sax-3(ky123)*, *daf-12(rh61)*, *daf-12(rh61rh411)*, *unc-40(e271)*, and *unc-40(e1430)*. *unc-40(e1430)* was marked with *dpy-5(e61)*. *unc-40(e271)* is a strong loss-of-function allele, while *unc-40(e1430)* is a null [6], [56]. Integrated strains were as follows: *unc-86::myr-GFP(kyIs262)* (gift from C. Bargmann, Rockefeller University, New York, NY) , *tph-1::GFP(mgIs42)* (gift from J. Sze, Albert Einstein College of Medicine, Yeshiva University, Bronx, NY), *lin-4::GFP(zaIs1)*, *lin-14::GFP(zaIs2)* and *lin-4 O/E(zaIs3)* [5], [43], [50], [76]. *lin-14::GFP(zaIs2)* was generated from the *lin-14::GFP* translational reporter strain *maEx166* [43], while *lin-4 O/E(zaIs3)* was generated from *lin-4 O/E #4* (Figure 1 and Figure 5). Integrations were performed using trimethylpsoralen and 365nm irradiation, and both strains were backcrossed at least three times prior to analysis.

To generate stable transgenic lines, experimental plasmids were co-injected into adult N2 animals with *myo-2::GFP* (5–10 ng/µl) or *rol-6* (75 ng/µl) injection markers as previously described [77]. The *myo-2::GFP* marker was used in injections of *lin-28::GFP* and *lin-28::GFPΔLCE*, and the *rol-6* marker was used for all other injections. Plasmid concentrations for injections were as follows: 10 ng/µl *lin-28::GFP(pVT218)* (gift from E. Moss, University of Medicine and Dentistry of New Jersey, Stratford, NJ [24], 5 ng/µl *lin-28::GFPΔLCE*, 1.5 ng/µl *unc-86::lin-4*, 1.5 ng/µl *unc-86::lin-4ΔMATURE*, and 1.5 ng/µl *unc-86::dsRED2*. The latter construct was used to tag the HSNs in lines expressing *unc-86::lin-4* or *unc-86::lin-4ΔMATURE*.

### Plasmid construction

The Invitrogen GeneTailor Site-Directed Mutagenesis Systemwas used during the preparation of *lin-28::GFPΔLCE* and *unc-86::lin-4ΔMATURE*, and the Invitrogen TOPO TA Cloning Kit for Sequencing was used to clone all PCR products. The LCE was removed from the *lin-28* 3′UTR in pVT218 through PCR using the mutagenic L28UTRM2F (5′-CCACCTACCTCCTCAAATTCTTTTTTTTTTC) and reverse L28UTR-R (5′- TTTGAGGAGGTAGGTGGTAGTATGGTT) primers. To generate the *unc-86::lin-4* construct, the *lin-4* hairpin and 80-nt flanking sequences were amplified by PCR from the pVTSal6 rescuing fragment (gift from V. Ambros, University of Massachusetts Medical School, Woucester, MA [19] using XMA-LIN4+ (5′-TCCCCCCGGGAATATAATAAATCTT) and NCO-LIN4- (5′-CATGCCATGGTACCATTTTATTGGA) primers, and the resulting product was inserted into pCR4.0-TOPO. A sequence-verified *lin-4::pCR4.0-TOPO* clone was digested with *Xma*I and *Nco*I, and the *lin-4* fragment was inserted into an *Xma*I/*Nco*I-cut construct containing a 5-kilobase *unc-86* promoter in pSM, a modified version of the pPD49.26 Fire vector (pSM-unc86; gift from C. Bargmann, Rockefeller University, New York, NY [53]). The 21-nt mature *lin-4* sequence from *lin-4::pCR4.0-TOPO* was removed by PCR using the mutagenic LIN4CON+ (5′- ATGCTTCCGGCCTGTGTGTACTATTGATGCT) and reverse LIN4CREV (5′- ACAGGCCGGAAGCATAAACTCATAAACCAA) primers. Once the deletion was verified, the truncated *lin-4* sequence was digested with *Xma*I and *Nco*I and sub-cloned into *Xma*I/*Nco*I-cut pSM-*unc-86*. dsRED2 was PCR-amplified from pdsRED2 (Clontech Catalog No. 632404) using NHE-RED+ (5′- CTAGCTAGCATGGCCTCCTCCGAGAA) and KPN-RED- (5′- GACTGGTACCTCACAGGAACAGGTGGT) primers. The resulting product was inserted into pCR4.0-TOPO, and DNA from a sequence-verified clone was digested with *Nhe*I and *Kpn*I and ligated to *Nhe*I/*Kpn*I-cut pSM-*unc-86*.

### Staging and identifying the HSNs

Staging was performed on the basis of gonad morphology, size, and as previously described [16]. Larvae in which distal tip cells had initiated their dorsal turn were scored as early L4. Animals with fully turned gonad arms with three or fewer embryos were categorized as young adult. Data from older adults were not included in this study. The HSNs were identified based on morphology, focal plane, and dorsal/ventral position as well as by analyzing patterns of axon outgrowth [11]. In cases where HSNs could not be conclusively identified based on these criteria, they were not scored.

### Microscopy and data analysis

Animals were anaesthetized in 5 mM levamisol directly on a slide (Figure 1A, wt L4 and adult; Figure 2C, *lin-28(lf)* early L3) or on 2–5% agarose pads. Imaging was performed on the Zeiss Axioplan 2 microscope equipped with a mercury arc lamp, using the Endow GFP bandpass (Chroma #41017) and/or HQ TRITC (Chroma #41002c) filter sets and the Zeiss Plan-Apochromat 100×/1.4 N.A. objective unless otherwise indicated. For quantitative studies, HSNs were first identified by DIC, and fluorescence images were then acquired with the Zeiss AxioCam MRm camera and the automated Zeiss AxioVision (v. 4.6) multidimensional acquisition module. Acquisition parameters were optimized to ensure signals fell as close as possible to the center of the camera's linear range, and were identical in cases where fluorescence intensity values were to be compared across samples. Image display was linear for all quantitative studies and was otherwise optimized in AxioVision (v. 4.6) or Adobe Photoshop 7.0. Average pixel intensity values were determined for the cell cytoplasm in *lin-28::GFP* strains and the nucleus in *lin-4::GFP* and *lin-14::GFP* strains. These regions were selected based on pixel intensity in the AxioVision AutoMeasure module, and separation lines were drawn when necessary. Since background subtraction did not alter trends observed in a representative experiment with the *lin-4::GFP(zaIs1)* reporter, it was not utilized during analysis of any images. Standard error of the mean was calculated for all average pixel intensity values, and the significance of the difference between two means was assessed with the two-sample *t*-test.

For the purposes of this study, an HSN process was scored as an axon if 1) it was the longest neurite and 2) it had extended ventrally and initiated an anterior (or rarely, posterior) turn or, for *lin-14(gf)* animals and strains depicted in Figure 6, it was at least three times the length of the HSN anterior/posterior cell diameter. If HSN axons could not be clearly resolved for any reason, they were not scored. Animals were designated as positive for HSN axon elongation or *tph-1::GFP* expression if the phenotype was observed in at least one of the two HSNs. The standard error of proportion was calculated for all proportional data, and the significance of the difference between proportions was determined using the two-sample *z*-test.

In the experiments described in Figure 6B, axons extending from the cells scored in Figure 6A were deemed misguided if they failed to grow initially at least one anterior/posterior cell diameter in the ventral direction before turning. In Figure 6D, axons scored in Figure 6C were categorized according to the direction in which they first extended at least one anterior/posterior cell diameter.

## Supporting Information

Figure S1 *lin-4* and its targets, *lin-14* and *lin-28*, temporally regulate *tph-1* expression. (A,B) Percentage of wild-type or mutant animals which expressed the integrated *tph-1::GFP* transgene in at least one HSN during L4 and adult stages. (A) At the restrictive temperature (23°C), *lin-14(lf)* mutant animals initiated precocious GFP expression at the L4 stage, and *lin-14(lf)* was sufficient to suppress the *lin-4(lf)* retarded phenotype in double *lin-4(lf)*; *lin-14(lf)* mutants. (B) *lin-28(lf)* animals displayed precocious *tph-1::GFP* expression, and *lin-28(lf)* suppressed the *lin-4(lf)* retarded phenotype. For (A), n≥50, and for (B), n≥100 for all conditions, and error bars represent standard error of proportion. (C) Representative images of L4- and adult-stage HSNs using DIC or fluorescence microscopy. At the L4 stage, GFP was not detectable in wild-type HSNs, and was precociously expressed in *lin-14(lf)* or *lin-28(lf)* HSNs. In the adult, GFP was present in the HSNs of wild-type animals but not in *lin-4(lf)* mutants. Arrowhead: HSN cell body. Scale bars represent 5 µm, and anterior is to the left and ventral is down.
(2.00 MB EPS)
